# Repair of non-lethal vascular injury caused by giant anteater (Myrmecophaga tridactyla) in Brazil

**DOI:** 10.1590/1677-5449.210081

**Published:** 2022-01-31

**Authors:** Vinicius Tadeu Ramos da Silva Grillo, Rodrigo Gibin Jaldin, William Wakasugui, Marcelo Sembenelli, Vidal Haddad

**Affiliations:** 1 Universidade Estadual Júlio de Mesquita Filho – UNESP, Faculdade de Medicina de Botucatu, Hospital das Clínicas, Botucatu, SP, Brasil.

**Keywords:** giant anteater, wild animals, vascular system injuries, vascular surgical procedures

## Abstract

The giant anteater is a mammal found in Central and South America. These animals have claws that can reach 6.5 centimeters in length, which they use to dig anthills to obtain food and for defense. We report the case of a 52-year-old male patient with a history of epilepsy who was taken unconscious to the emergency room due to injuries to his right arm caused by an anteater. He underwent surgical exploration to investigate suspected vascular trauma, revealing a combined (arterial and venous) injury of the brachial vessels, which were repaired. He recovered well and was discharged on the second postoperative day. During outpatient follow-up he continued to improve, with no neurological or vascular sequelae.

## INTRODUCTION

**Figure 1:**
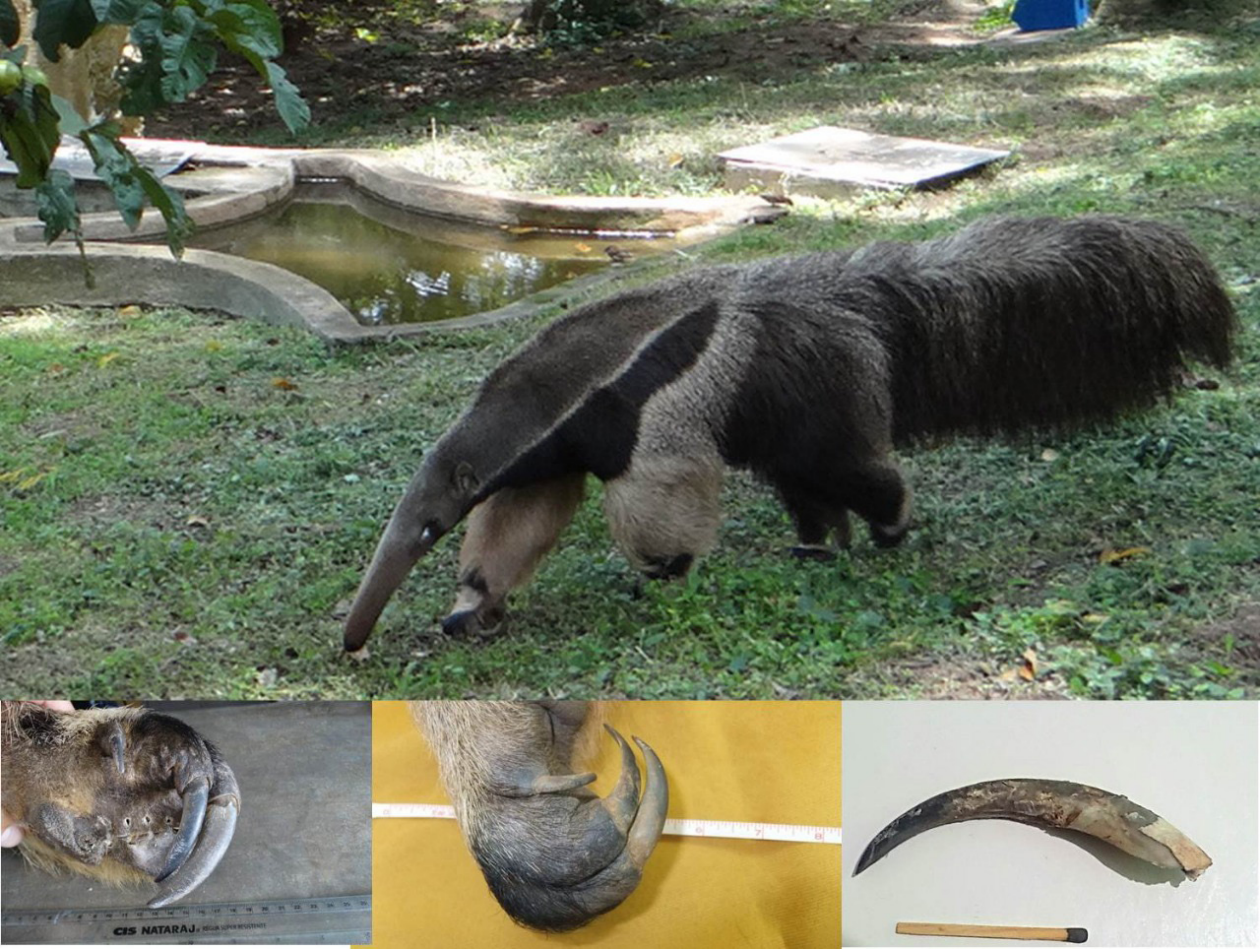
Giant anteater (*Myrmecophaga tridactyla*). Inserts: fore paw claws. Photographs from the personal archives of Prof. Vidal Haddad Jr.

The giant anteater is a toothless mammal found in Central and South America that can weigh up to 40 kg and measure more than 2 m in length (Figure 1). It has an acute sense of smell, but its sight and hearing are weak. The claws on its fore paws can grow up to 6.5 cm in length and are used to obtain food by digging up ant hills and termite mounds (Figure 1). The animal can also use them as tools for defense^1^. 

Increasing urbanization, expansion of agriculture, predatory hunting, fires, and road kills (a consequence of its poor sight) have contributed to decline in the species’ populations^1^. Giant anteaters are not habitually aggressive, but may adopt a defensive position if they feel threatened, rearing up on their hind legs to employ the powerful claws on their fore paws. It is very rare for them to attack humans and the most common cases involve researchers or people who threaten or harm them. Their sharp claws can cause serious injuries, including lacerations, hemorrhages^2^, and even death^2^^,^ 3. 

This study was duly assessed and approved by a Research Ethics Committee (CAAE 45868021.4.0000.5411, ruling number 4.698.941).

## CASE REPORT

**Figure 2:**
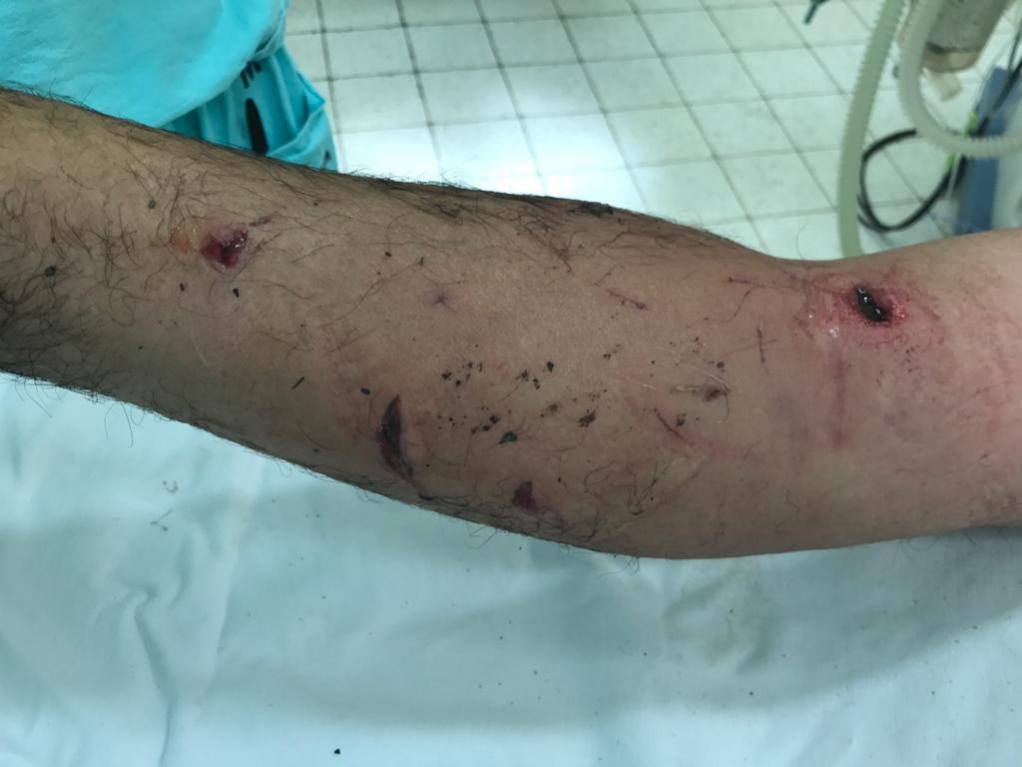
Blunt and penetrating lesions to the arm and forearm of the right upper limb.

A 52-year-old male patient from Torre de Pedra, SP, Brazil, was brought to the emergency service of a tertiary hospital by his brother. His companion stated that he had found the victim unconscious in a rural area after being attacked by a giant anteater while hunting. There were visible injuries to the patient’s right upper limb. He was admitted to the emergency room at the Hospital das Clínicas, without a spine board or cervical collar. Initial care was provided as recommended by Advanced Trauma Life Support^4^. Airways were patent, with no stridor, bleeding, or foreign bodies in the oral cavity. The patient was breathing spontaneously, with no respiratory distress, respiratory rate of 16 breaths per minute, expansibility maintained and symmetrical, with audible and symmetrical vesicle murmurs, percussion provoking clear pulmonary sounds, and peripheral oxygen saturation of 97%. He was normotensive, with blood pressure of 150x90 mmHg, and heart rate of 74 beats per minute. His abdomen was free of visible injuries, ecchymosis, or hematoma; flat, flaccid, and normotensive, with digestive sounds present and without painful sudden decompression. The pelvis was stable. He was scored three on the Glasgow Coma Scale (no verbal response = one point, no motor response = one point, no eye opening = one point), pupils were equal but reacted slowly to light. On admission, the patient had blunt and penetrating traumas to the right upper limb, without active bleeding, and the following injuries were described: a 2 cm lesion on the anterior aspect of the distal third of the forearm; a 4 cm lesion on the anteromedial aspect of the mid third of the forearm, exposing the subcutaneous tissue; and a 3 cm lesion in the antecubital fossa (Figure 2).

During the vascular examination at admission, the right upper limb had a temperature gradient and was cold up to the elbow, but with no cyanosis or livedo.

After additional investigation, the team was informed that the patient had a history of poorly-controlled epilepsy and it was hypothesized that a non-convulsive grand mal seizure was the cause of his reduced state of consciousness.

Benzodiazepine was administered, the patient’s state of consciousness improved, and from this point on he remained awake, conscious, and oriented. Results of laboratory tests conducted at admission included hemoglobin at 12.04 g/dL (reference range [RR] 14-18 g/dL), white blood cell count of 10,950/mm^3^ (RR 4,000-11,000/mm^3^), platelets at 158,000/µL (RR 140,000-440,000/µL), electrolytes and coagulogram both normal, and creatine phosphokinase of 283 U/L (RR 32-294 U/L).

**Figure 3:**
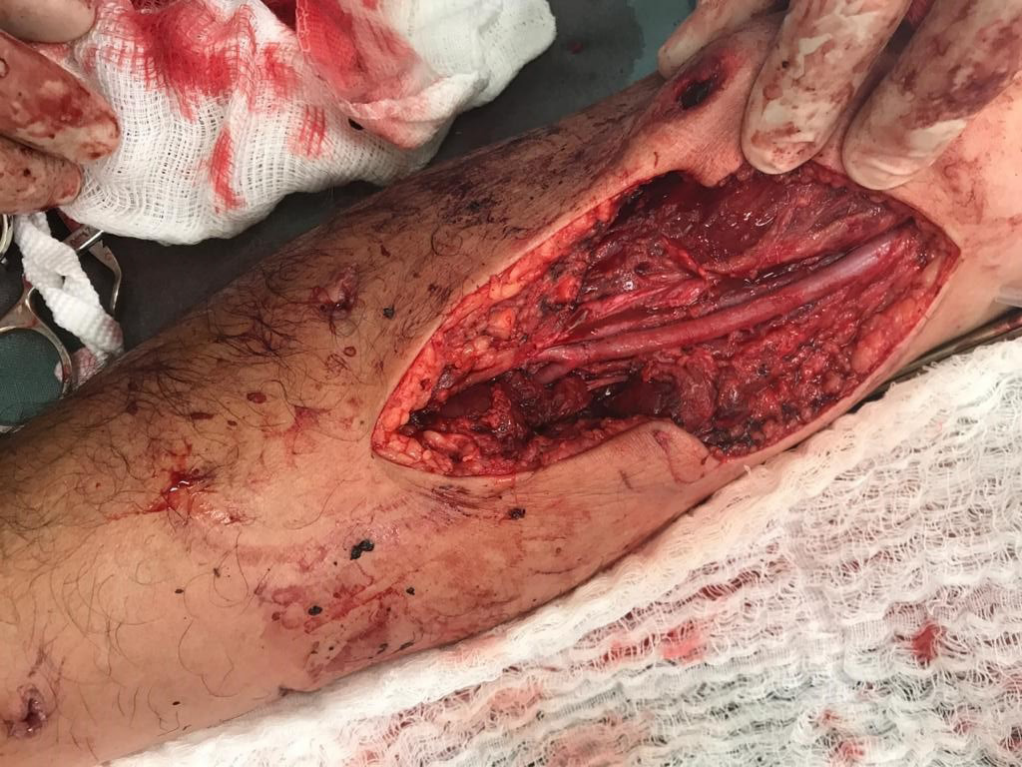
Result of surgical dissection, with exposure of the brachial artery and its bifurcation into the radial and ulnar arteries. Note the arteriorrhaphy of the brachial artery, performed after embolectomy with a Fogarty catheter.

The vascular surgery team’s specialist assessment found no palpable radial or ulnar pulses, identifying a biphasic flow pattern on Doppler velocimetry with direct Doppler sonar. Vascular echography did not show rupture of the walls of the brachial, radial, or ulnar arteries, but the acoustic window onto one segment of the brachial artery was compromised by trauma to soft tissues. Additionally, the radial and ulnar arteries exhibited monophasic wave patterns with low amplitude. In view of these findings, the team decided to conduct surgical exploration via a bayonet incision in the antecubital fossa, providing ample exposure of the brachial artery and its bifurcation into the radial and ulnar arteries. After the incision had been made, hematoma was identified and drained, revealing that the brachial artery had no pulse and showed signs of segmental contusion/dissection from hematoma to the anterior wall, probably caused by contusion. In addition to the vascular injury, there was moderate laceration of the adjacent musculature, but with no evidence of nerve or tendon damage during exploration. The team proceeded with arteriotomy, finding intraluminal thrombus. Embolectomy was then performed with a Fogarty catheter, removing clots and restoring pulsating flow. Total section of the radial veins was also identified and treated by simple ligature (Figure 3). At the end of the procedure, the patient had 4+/4+ radial and ulnar pulses.

While still in hospital, the patient was given saline, anti-rabies vaccine, and anti-tetanus vaccine, as instructed by the infectology service. He had good clinical progress and was discharged from hospital on the second postoperative day with a prescription for 100 mg aspirin once a day and told to consult the neurology service for follow-up. At outpatients follow-up, the patient still had palpable distal pulses, had no vascular or neurological complaints, and was free from pain, although he had a mild infection of the operating wound and was treated with ciprofloxacin and clindamycin for 14 days. At his most recent consultation with the vascular surgery team, in the 12th postoperative month, the patient’s operating wound was completely healed, his radial pulse was palpable, he had no significant neurological or musculoskeletal sequelae, and he was discharged from follow-up by the specialty.

## DISCUSSION

Injuries caused by anteaters when in the defense position are rare and the victims are generally professionals who study them in the wild or are responsible for caring for them in zoos. These animals tend to avoid contact with humans, but because of their weak hearing and sight, avoidance is not always possible, which increases the likelihood of accidents^2^. 

Treatment of the injuries caused by anteaters is based on the principles of trauma surgery. Control of hemorrhage and rapid transport to an appropriate center are essential for a good outcome. The anteater’s claws can cause serious skin injuries and are able to reach the majority of organs and cause deep hemorrhages that are difficult to control, with risk of death from hemorrhagic shock^3^. 

It is necessary to prescribe anti-rabies vaccine because, since the anteater is a mammal, it is susceptible to contracting the rabies virus. Management of injuries caused by any type of wild animal includes meticulous cleaning of the site and wide-spectrum antibiotic therapy, primarily targeting coverage of gram-positive microorganisms, due to the high risk of related secondary infections^2^. 

In two prior reports of deaths caused by giant anteaters, the cause of death was femoral artery hemorrhage, which is reinforced by observation of the present case and furnishes evidence that the serious accidents caused by these animals are related to vascular injuries and intense hemorrhage. In the case described here, there was a combined (arterial and venous) vascular injury. The team chose arterial revascularization of the ischemic limb with brachial artery embolectomy with a Fogarty catheter and ligature of the radial veins, since there was no benefit to be gained from venous reconstruction in this situation.

Contact with wild animals should be avoided and human contact with them in their own habitats should be calculated. People should be aware that all animals defend themselves in situations of stress, especially if chased or hunted, as in the case described^2^.

## References

[1] Braga FG (2010). Ecologia e comportamento de tamanduá-bandeira.

[2] Haddad V, Reckziegel GC, Domingos G, Pimentel FL (2014). Human death caused by a giant anteater (Myrmecophaga tridactyla) in Brazil. Wilderness Environ Med.

[3] Haddad VH, Nunes JF (2016). Report of a new human death caused by a giant anteater in Brazil. Wilderness Environ Med.

[4] ATLS Subcommittee (2013). Advanced trauma life support (ATLS®). J Trauma Acute Care Surg.

