# Supplementary material for: Repair of non-lethal vascular injury caused by giant anteater (Myrmecophaga tridactyla) in Brazil
Source: J Vasc Bras. 2022 Jan 31;21:e20210081. doi: 10.1590/1677-5449.210081 (PMC8958431; doi:10.1590/1677-5449.210081)
Supplement: Translation

# Translation: Reparo de lesão vascular não letal causada por tamanduá-bandeira (*Myrmecophaga tridactyla*) no Brasil

**Authors:** Vinicius Tadeu Ramos da Silva Grillo, Rodrigo Gibin Jaldin, William Wakasugui, Marcelo Sembenelli, Vidal Haddad

**Affiliations:** 1 Universidade Estadual Júlio de Mesquita Filho – UNESP, Faculdade de Medicina de Botucatu, Hospital das Clínicas, Botucatu, SP, Brasil.

**Keywords:** tamanduá-bandeira, animais selvagens, lesões do sistema vascular, procedimentos cirúrgicos vasculares

## Abstract

O tamanduá-bandeira é um mamífero encontrado na América Central e na América do Sul. Esse animal possui garras que podem chegar a 6,5 cm de comprimento, utilizadas para escavar formigueiros e obter alimento, além de servir para sua defesa. Relatamos o caso de paciente masculino de 52 anos, com histórico de epilepsia, que foi levado desacordado ao pronto-socorro, devido a lesões no seu braço direito causadas por um tamanduá. Frente à suspeita de trauma vascular, o paciente foi submetido a exploração cirúrgica, que evidenciou uma lesão combinada de vasos braquiais, submetida a reparo. Apresentou boa evolução do quadro, recebendo alta hospitalar no segundo dia de pós-operatório e, no seguimento ambulatorial, evoluiu sem sequelas neurológicas ou vasculares.

## INTRODUÇÃO

O tamanduá-bandeira é um mamífero desdentado encontrado na América Central e na América do Sul que pode pesar até 40 kg e medir mais de 2 m de comprimento (Figura 1). Seu olfato é aguçado, mas a visão e a audição são reduzidas. As unhas das patas dianteiras podem chegar a 6,5 cm de comprimento e são utilizadas para obtenção de alimento, através da escavação de formigueiros e cupinzeiros (Figura 1). Elas podem ainda ser instrumentos de defesa do animal^1^.

**Figura 1:** Tamanduá-bandeira (*Myrmecophaga tridactyla*). No detalhe: unhas das patas dianteiras. Fotografias do acervo pessoal do Prof. Vidal Haddad Jr.

A crescente urbanização, expansão da agricultura, caça predatória, queimadas e atropelamentos (devido à visão deficiente) têm contribuído para o declínio das populações dessa espécie^1^. Os tamanduás-bandeira não costumam ser agressivos, mas podem assumir posição de defesa quando se sentem ameaçados, ficando em posição vertical para utilizar as poderosas garras das patas anteriores. O ataque a seres humanos é muito raro e envolve, com maior frequência, pesquisadores ou indivíduos que ameaçam ou agridem esses animais. As garras afiadas podem provocar graves lesões, incluindo lacerações, hemorragias^2^ e até morte^2^^,^ 3.

Este estudo foi devidamente avaliado e aprovado pelo Comitê de Ética em Pesquisa (CAAE 45868021.4.0000.5411 e parecer número 4.698.941).

## RELATO DE CASO

Paciente masculino de 52 anos, procedente de Torre de Pedra (SP), deu entrada no serviço de emergência de um hospital terciário, trazido pelo irmão. O acompanhante referiu ter encontrado a vítima inconsciente em ambiente rural devido ao ataque de um tamanduá-bandeira enquanto desenvolvia atividade de caça, com lesões identificáveis no membro superior direito. O paciente foi admitido na sala de emergência do Hospital das Clínicas, sem prancha rígida ou colar cervical. Realizou-se atendimento conforme preconizado no *Advanced Trauma Life Support* 4. As vias aéreas encontravam-se pérvias, sem estridor, sangramento ou corpo estranho na cavidade oral; além disso, o paciente apresentava respiração espontânea sem taquidispineia, frequência respiratória de 16 incursões por minuto, expansibilidade preservada e simétrica, com murmúrios vesiculares audíveis e simétricos, percussão com som claro pulmonar e saturação periférica de oxigênio de 97%. Paciente normotenso, com pressão arterial de 150x90 mmHg e frequência cardíaca de 74 batimentos por minuto. Abdome sem lesões aparentes, equimoses ou hematomas; plano, flácido, normotenso, com ruídos hidroaéreos presentes, sem descompressão brusca dolorosa. Pelve estável. Escala de Coma de Glasgow de três (ausência de resposta verbal = um ponto, ausência de resposta motora = um ponto, ausência de resposta ocular = um ponto), pupilas isocóricas e bradirreagentes. À admissão, o paciente apresentava ferimentos corto-contusos no membro superior direito, sem sangramento ativo, com os seguintes ferimentos descritos: lesão de 2 cm em face anterior do terço distal do antebraço; lesão de 4 cm em face anteromedial do terço médio do antebraço, com exposição do tecido celular subcutâneo; e lesão de 3 cm em fossa antecubital (Figura 2).

**Figura 2:** Lesões corto-contusas no braço e antebraço do membro superior direito.

Ao exame vascular na entrada, o membro superior direito apresentava gradiente térmico com frialdade até o cotovelo, porém sem cianose ou livedo.

Após investigação complementar, a equipe foi informada de que o paciente apresentava histórico de epilepsia mal controlada, com hipótese de estado de mal epiléptico não convulsivo como etiologia do rebaixamento do nível de consciência.

Foi administrado benzodiazepínico, com recuperação do nível de consciência, mantendo-se, a partir de então vígil, consciente e orientado. Apresentava, aos exames laboratoriais de admissão, hemoglobina 12,04 g/dL [valor de referência (VR) 14-18 g/dL], leucócitos 10.950/mm^3^ (VR 4.000-11.000/mm^3^), plaquetas 158.000/µL (VR 140.000-440.000/µL), eletrólitos e coagulograma normais, e creatinofosfoquinase 283 U/L (VR 32-294 U/L).

À avaliação especializada da equipe da cirurgia vascular, não havia pulsos radial ou ulnar palpáveis, identificando-se fluxo de padrão bifásico à dopplerfluxometria com sonar Doppler direito. A ecografia vascular não evidenciou descontinuidade parietal nas artérias braquial, radial ou ulnar, porém o segmento da artéria braquial apresentava janela acústica prejudicada, devido a trauma de partes moles. Além disso, as artérias radial e ulnar mostravam padrão de onda monofásica e de baixa amplitude. Diante dos achados, optou-se pela realização de exploração cirúrgica por incisão em baioneta na fossa antecubital, com exposição ampla da artéria braquial e sua bifurcação em artérias radial e ulnar. Após a incisão, foi identificado hematoma e, após drenagem deste, a artéria braquial apresentava-se sem pulsatilidade e com sinais de contusão/dissecção segmentar por hematoma na parede anterior, provavelmente devido à contusão. Associada à lesão vascular, havia laceração moderada da musculatura adjacente, porém sem evidência de lesão nervosa ou tendinosa durante a exploração. Foi realizada arteriotomia, com identificação de trombo intraluminal. Dessa forma, procedeu-se a embolectomia com cateter de fogarty e retirada de coágulos, retornando o fluxo pulsátil. Identificou-se ainda secção completa das veias radiais, a qual foi tratada por ligadura simples (Figura 3). Ao final do procedimento, o paciente evoluiu com pulsos radial e ulnar 4+/4+.

**Figura 3:** Resultado da dissecção cirúrgica, com exposição da artéria braquial e de sua bifurcação em artérias radial e ulnar. Nota-se arteriorrafia de artéria braquial, após embolectomia com cateter de fogarty.

Durante a internação, foram administrados soro, vacina antirrábica e vacina antitetânica, conforme orientação do servico de infectologia. O paciente apresentou boa evolução clínica e recebeu alta hospitalar no segundo dia de pós-operatório, sendo prescrito ácido acetilsalicílico 100 mg uma vez ao dia e orientado acompanhamento com serviço de neurologia. No retorno ambulatorial, o paciente mantinha pulsos distais palpáveis, sem queixas álgicas, vasculares ou neurológicas, porém com infecção leve de ferida operatória, sendo tratado com ciprofloxacino associada à clindamicina por 14 dias. Em última avaliação pela equipe da cirurgia vascular no 12º mês de pós-operatório, o paciente apresentava a ferida operatória totalmente cicatrizada, com pulso radial palpável e sem sequelas neurológicas ou osteomusculares importantes, recebendo alta do seguimento pela especialidade.

## DISCUSSÃO

As lesões causadas pelos tamanduás, quando em posição de defesa, são raras e geralmente ocorrem em profissionais que estudam animais na natureza ou que são responsáveis pelos cuidados dessa espécie em zoológicos. Esses animais costumam evitar o contato com os humanos; porém, devido à sua visão e audição reduzidas, isso nem sempre é possível, aumentando a chance de ocorrer acidentes^2^.

O tratamento de lesões causadas por esse animal segue as bases da cirurgia do trauma. O controle da hemorragia e a agilidade no transporte para um centro adequado são fundamentais para um bom desfecho. As garras do tamanduá podem causar sérias lesões na pele, com a possibilidade de atingir a maioria dos órgãos e causar hemorragias profusas e de difícil controle, com risco de óbito por choque hemorrágico^3^.

A indicação de vacinação antirrábica é necessária, pois, por ser um mamífero, o tamanduá está sujeito a adquirir o vírus da raiva. A conduta adotada para lesão por qualquer animal selvagem é a limpeza rigorosa do local e uso de antibioticoterapia de amplo espectro, principalmente para cobertura contra microrganismos gram-positivos, devido ao alto risco de infecções secundárias associadas^2^.

Em dois relatos anteriores de mortes causadas por tamanduás-bandeira, a causa foi hemorragia da artéria femoral, o que é reafirmado pela observação deste caso e evidencia que os acidentes graves causados por esses animais estão associados a lesões vasculares e hemorragia intensa. Como no caso descrito, houve lesão vascular combinada (arterial e venosa), optando-se pela revascularização arterial do membro isquêmico por embolectomia com cateter de fogarty da artéria braquial e pela ligadura das veias radiais, pois não haveria benefício de reconstrução venosa nessa situação.

Os animais selvagens devem ser evitados, e o contato humano em seus ambientes deve ser calculado, com as pessoas tendo conhecimento de que qualquer animal se defende em situação de estresse, especialmente quando perseguido ou caçado, como no caso relatado^2^.
